# Elastic-net regularization approaches for genome-wide association studies of rheumatoid arthritis

**DOI:** 10.1186/1753-6561-3-s7-s25

**Published:** 2009-12-15

**Authors:** Seoae Cho, Haseong Kim, Sohee Oh, Kyunga Kim, Taesung Park

**Affiliations:** 1 Interdisciplinary Program in Bioinformatics, Seoul National University, Kwan-ak St. 599, Kwan-ak Gu, Seoul, South Korea 151-741, Republic of Korea; 2 Department of Electrical and Electronic Engineering, Imperial College London, SW7 2AZ, UK; 3 Department of Statistics, Seoul National University, Kwan-ak St. 599, Kwan-ak Gu, Seoul, South Korea 151-741, Republic of Korea

## Abstract

The current trend in genome-wide association studies is to identify regions where the true disease-causing genes may lie by evaluating thousands of single-nucleotide polymorphisms (SNPs) across the whole genome. However, many challenges exist in detecting disease-causing genes among the thousands of SNPs. Examples include multicollinearity and multiple testing issues, especially when a large number of correlated SNPs are simultaneously tested. Multicollinearity can often occur when predictor variables in a multiple regression model are highly correlated, and can cause imprecise estimation of association. In this study, we propose a simple stepwise procedure that identifies disease-causing SNPs simultaneously by employing elastic-net regularization, a variable selection method that allows one to address multicollinearity. At Step 1, the single-marker association analysis was conducted to screen SNPs. At Step 2, the multiple-marker association was scanned based on the elastic-net regularization. The proposed approach was applied to the rheumatoid arthritis (RA) case-control data set of Genetic Analysis Workshop 16. While the selected SNPs at the screening step are located mostly on chromosome 6, the elastic-net approach identified putative RA-related SNPs on other chromosomes in an increased proportion. For some of those putative RA-related SNPs, we identified the interactions with sex, a well known factor affecting RA susceptibility.

## Background

Recently, genome-wide association studies (GWAS) have become a promising new tool for deciphering the genetics of complex diseases, which are usually polygenic and affected by gene-by-environmental interactions. Because it can be more powerful to scan multiple markers jointly in detecting disease-related genes, various multiple-marker approaches have been or can be used in GWAS [1-4]. Examples include logic regressions [2] and classification and regression trees [3]. Due to their sequential selection processes, these methods may miss the overall correlation structure of the genes. Another example is random forest [4], based on which true disease-causing genes can be hidden due to other genes; the identification result may not be robust.

In this study, we propose a simple stepwise procedure that employs the elastic-net regularization-based approach [5] to take the overall correlation structure of single-nucleotide polymorphisms (SNPs) into account when selecting disease-causing genes automatically in GWAS. Because the elastic net imposes on a combination of lasso and ridge penalties [6,7], it provides a more reproducible prediction than using multiple regression, especially when there are highly correlated predictors (e.g., SNPs in high linkage disequilibrium). Our approach consists of two main steps, called the screen step and the elastic-net step. At the screen step, we eliminate most of noise SNPs via single-marker association tests, and select the largest number of candidate SNPs that can be analyzed by the elastic-net approach at the next step. At the elastic-net step, putative disease-causing SNPs are jointly identified based on multiple logistic regressions with the screened SNPs via the elastic net. Interactions between SNP and non-genotypic factor (e.g., sex) can also be examined.

The proposed approach was applied to the rheumatoid arthritis (RA) case-control dataset of Genetic Analysis Workshop 16 (GAW16). RA is a complex disease with a moderately strong genetic component. It is generally known that females are at a higher risk than males and the mean onset of disease is in the fifth decade. Many studies have implicated the HLA region on chromosome 6p21, with consistent evidence for several DR alleles contributing to risk [8]. Among the non-HLA loci, *PTPN22 *on chromosome 1p13, a gene coding for protein tyrosine non-receptor22, is considered as a strong candidate RA-susceptibility gene [9]. Recently, a functional SNP in this *PTPN22 *gene was reported to be associated with RA [10]. There remains much to learn about the genetic susceptibility for RA, including possible gene-by-environmental interactions.

## Methods

### Genotype data and sample

The RA data from GAW16 included 545,080 SNPs genotyped by Illumina (550 k chip) along with covariates for 908 cases and 1260 controls. We adjusted population stratification using the computer program Eigenstrat [11] by excluding 20 outliers from the samples. Also, the samples showing sex matching error were filtered [12]. We excluded SNPs with >10% missing genotype, with minor allele frequencies <5%, and/or with *p *< 0.001 from Hardy-Weinberg equilibrium tests. As a result, 474,499 SNPs passed our quality control filters and were used in the proposed stepwise analyses.

### Step 1: Screening SNPs via single-marker association tests

For each single SNP, the disease association is tested using the following logistic regression model adjusted by sex, under the additive mode of inheritance:

where *π *represents the probability of getting the disease. Among the SNPs showing the strongest associations, we select the largest number of SNPs that can be analyzed in the penalized logistic regression via the elastic net at the next step. This screening step is needed to address the computational limitation when applying the penalized logistic regression via the elastic net to multiple SNPs.

### Step 2: Penalized logistic regression models via the elastic net

In this step, putative disease-causing SNPs are identified via elastic-net-based variable selection. The elastic-net method is particularly useful when the number of highly correlated predictor variables (*p*) is much larger than the sample size (*N*). The elastic-net regularization approach solves the following problem:

where the elastic-net penalty is defined as

The elastic-net penalty creates a useful a compromise between the ridge-regression penalty (*α *= 0) [9] and the lasso penalty (*α *= 1) [10]. The elastic net with *α *= 1 - *ε *for some small *ε *> 0 performs much like the lasso, but is robust to extreme correlations among predictor variables. Moreover, the elastic net does both shrinkage and automatic variable selection simultaneously. The choice of the regularization parameter (*λ*) is critical to selecting important variables with accurate estimation. Tuning parameters *α *and *λ *are usually selected to minimize mean-squared prediction error based on cross-validations (e.g., 5-fold).

Because the effect of genotype variations (i.e., SNPs) on disease status can be modified by other factors (in our study, sex), we consider the following multiple logistic regression models to examine the SNP main effects (M1) and also interaction effects of SNPs with sex (M2).

where *π *represents the probability of getting the disease. When M1 is used with the elastic-net penalties, the SEX variable is not penalized to adjust the sex effect in selecting SNP main effects. Note that main effect terms of both SEX and SNPs are not penalized when examining the SNP-by-sex interactions in M2. In this study, we use a library 'glmnet' in R statistical package http://www.r-project.org to conduct the penalized logistic regressions via the elastic-net.

## Results

### Single-marker associations

**Figure 1:**
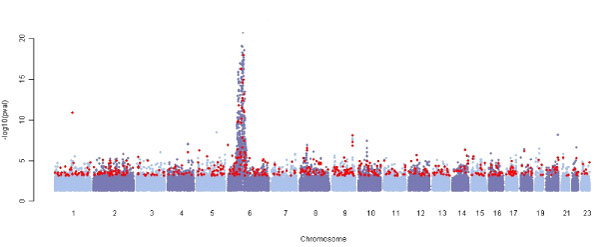
**Genome-wide scan for RA-SNP association**. The *p*-values < 0.05 from single SNP association tests were plotted in -log_10 _scale against chromosomal positions of the corresponding 48,366 SNPs. Blue and light blue were used to distinguish chromosomes. Red indicates potential RA-related SNPs that were identified by fitting the penalized logistic regression model (M1) via elastic-net using top 3000 of those 48,366 SNPs.

The single-marker association test was conducted for each SNP, and 48,336 SNPs showed *p*-values below 0.05 (Figure 1). Some SNPs are in *HLA-DRB1 *and *PTPN22*, which were already known to be RA-susceptibility genes [8-10]. Among the 48,336 SNPs, we chose the top 1000, 2000, and 3000 significant SNPs for Step 2.

### Main effect analysis via elastic-net (M1)

**Figure 2:**
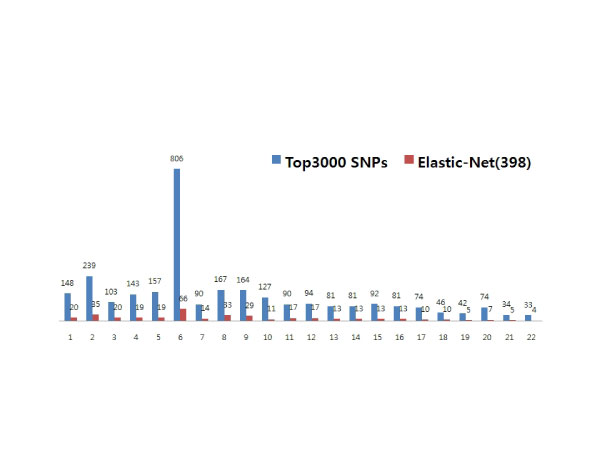
**Distributions of top 3000 screened SNPs vs. 398 potential RA-related SNPs across chromosomes**. For each chromosome, blue bars represent the number of SNPs that were selected as top 3000 SNPs via single SNP association tests at Step 1; and red bars represent the number of potential RA-related SNPs that were identified at Step 2 by fitting penalized logistic regression model (M1) via elastic-net using the top 3000 screened SNPs.

We applied the model M1 via the elastic net to top 1000, 2000, and 3000 SNPs selected at the first step. Among top 1000 SNPs, 250 SNPs were identified with main effects as putative RA-related SNPs while 360 SNPs were detected among the top 2000 and 398 SNPs among the top 3000. Those with the ten largest main effects are listed in Table 1. The resulting putative RA-related SNPs are displayed across the whole genome in Figure 1. Across the screening choices, 81 SNPs were commonly selected. Among those SNPs, 23 SNPs were identified also from single-marker association analyses after 5% Bonferroni multiplicity correction, and (except three SNPs) are located on chromosome 6. For examples, rs2395175 and rs660895 in *HLA-DRB1 and HLA-DRA on chromosome 6 *had *p*-values of 1.08 × 10^-87 ^and 7.16 × 10^-90^, respectively, from single-marker association test. However, 58 overlapping SNPs that were not identified from single-marker association analyses were found on various chromosomes. Some SNPs are located on known genes, such as *AMFR*, *ANKRD35*, *ECT2*, *TARBP1*, *ZFP92*, and *ZFPM2*. For instance, rs2440468 is located in AMFR (autocrine motility factor receptor) gene on chromosome 16. AMF secretion and receptor levels are closely related to RA as well as tumor malignancy [13]. Note that RA-susceptibility odds ratios (ORs) of AG and GG against AA were 0.78 and 0.57, respectively, for this SNP. However, rs2440468 had a *p*-value = 5.74 × 10^-5 ^for single-marker association test. While the evidence for single-marker based association at chromosome 6 with RA has been previously identified by numerous studies [1], our results indicate that putative RA-related SNPs were also distributed across several other regions outside of the chromosome 6 (Figure 2).

**Table 1:** RA-related SNPs identified with ten largest main effects via the elastic-net method (M1)

SNP	Chromosome^a^	Coefficient^b^
Top 1000		
rs6903608	6	-0.3413
rs2395185	6	0.3285
rs11686264	2	-0.3284
rs6981223	8	-0.31
rs10948693	6	-0.2813
rs9727917	1	-0.2806
rs2440468	16	-0.2736
rs4499874	5	0.2714
rs9275595	6	0.2641
rs7970893	12	-0.2492
Top 2000		
rs2395175	6	0.2522
rs6903608	6	-0.2299
rs10094729	8	-0.166
rs2101613	10	-0.1613
rs6910071	6	0.1529
rs660895	6	0.1522
rs9277554	6	-0.1468
rs12203592	6	-0.1401
rs2578240	9	0.1356
rs9275572	6	-0.1353
Top 3000		
rs2395175	6	0.3532
rs660895	6	0.2302
rs9275572	6	-0.219
rs10094729	8	-0.1972
rs6903608	6	-0.1889
rs3873444	6	-0.1403
rs7970893	12	-0.1321
rs234592	14	-0.1316
rs10789176	1	-0.125
rs9275601	6	-0.1221

^a^Chromosome where SNP is located.

^b^Coefficient representing size and direction of SNP main effect.

### Interaction analysis with sex via elastic-net (M2)

To investigate SNPs with effects on RA-susceptibility that varied across sexes, we performed interaction analysis (M2) with the putative RA-related SNPs from M1 for each screening choice (i.e., top 1000, 2000, and 3000). We identified 71 SNPs and 132 SNPs with the SNP-by-sex interaction for each choice of top 1000 and top 2000, while 105 SNPs showed interactions for top 3000 choice. Those with five largest interactions effects are summarized in Table 2. For each sex, we investigated RA-susceptibility OR of each genotype against major-allele homozygote. For example, rs2044750 showed heterozygote OR of 1.12 and 1.71 for female and male, respectively. The OR for AA is 1.37 for female and 2.37 for male. This SNP is located in nuclear factor of activated T cell 1 (*NFATc1*), a transcription factor on chromosome 18, which has recently been shown to be related to osteoporosis, bone metastasis, and rheumatoid arthritis [14]. Note that rs2044750 showed a non-small *p*-value of 0.00041 at single-marker association test. Note that ten SNPs overlapped across the screening choices. Out of ten SNPs, we found six SNPs in known genes, such as *C19orf2*, *CUGBP2*, *ECT2*, *TBC1D8*, and *WNT3*.

**Table 2:** RA-related SNPs identified with sex-by-SNP interaction via the elastic-net method (M2)

SNP	Chromosome^a^	Coefficient^b^
Top 1000		
rs2858870	6	-0.329
rs9727917	1	-0.2572
rs10184573	2	-0.2347
rs10514911	17	0.2314
rs11703151	22	0.2077
Top 2000		
rs6903608	6	-0.538
rs1217675	8	0.5188
rs560271	17	-0.5169
rs201119	10	-0.4943
rs2044750	18	-0.4812
Top 3000		
rs3873444	6	-0.3573
rs948195	11	-0.3233
rs2579088	12	0.3063
rs13277113	8	0.303
rs12407970	1	0.2787

^a^Chromosome where SNP is located.

^b^Coefficient representing size and direction of SNP main effect.

## Discussion

We have proposed a simple stepwise approach that employs the multiple logistic regression model with the elastic-net penalties to detect disease-causing genes across a whole genome in GWAS. The elastic-net method using both lasso and ridge penalties has several advantages in identifying disease-causing SNPs jointly in GWAS. First, automatic variable selection and continuous shrinkage can be simultaneously performed. Second, it can select groups of many highly correlated SNPs, which may cause a multicollinearity problem in classical multiple linear regressions. Third, the shrinkage feature of the elastic net enables us to include all the interaction terms between SNPs and non-genotypic factors as well as SNP main effects into a model. Also, rather than searching for potential SNPs along the entire chromosome directly, our approach provides an efficient search by using a multi-step procedure to handle the extremely large number of potential SNP patterns in GWAS.

Although most putative RA-related SNPs were found in chromosome 6, we also identified additional susceptibility genes in other chromosomes. Our findings need to be replicated in an independent dataset or to be functionally validated in the future in order to declare the biological significance. There is disagreement in results across the screening choices. There are possible causes that result in this discrepancy. First, the missing data caused large differences in the results. We removed some samples and SNPs to make datasets complete because the elastic-net regression method we employed does not allow for missing data. So the three datasets according to the screening choices ended up with different sample sizes. The difference in sample size was large in the previous analysis, and we tried to make the sample sizes similar in the updated analysis. Even though the previous analysis had a similar sample size, there are about only 70% overlapping samples, as shown below. This explains why we had more common SNPs in the updated results. This missing data problem would be avoided by using a proper imputation method for missing data. Second, depending on the correlation structures among SNPs, the elastic-net regression method may provide different results because it considers the correlation structure when selecting variables.

## List of abbreviations used

GAW16: Genetic Analysis Workshop 16; GWAS: Genome-wide association study; OR: Odds ratio; RA: Rheumatoid arthritis; SNPs: Single-nucleotide polymorphisms

## Competing interests

The authors declare that they have no competing interests.

## Authors' contributions

HK and SO participated in statistical analysis. SC participated in the design of the study, performed the statistical analysis, and drafted the manuscript. KK and TP conceived of the study, and participated in its design and coordination and helped to draft the manuscript. All authors read and approved the final manuscript.
